# 

**Published:** 1891-08-01

**Authors:** 


PRICE ONE PENNY.
No. 253, Vol. X.—August 1st, 1891.
[Registered at the General Post Office as a Newspaper for transmission in the United Kingdom and Abroad.]

WITH EXTRA NURSING SUPPLEMENT.
Per Annum, 6s. 6d.; post free.
Monthly Parts, 6d.; post free 8d.
Ditto per Annum, 8s., post free.

### THE HOSPITAL

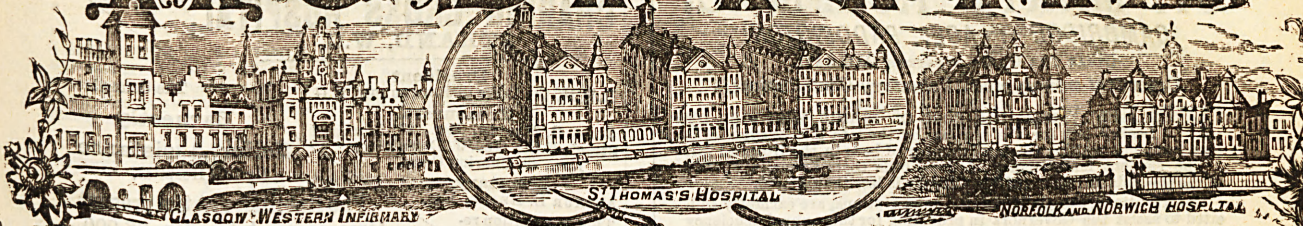

A WEEKLY JOURNAL OF
Science, Medicine, Nursing and Philanthropy.

SATURDAY, AUGUST 1st, 1891.

| | |
|---|---|
| **The Princess and the Second Thousand** | 205 |
| **Passing Topics.**—The Beginning of the End | 206 |
| **The Doctor's Armchair.**—Husbands | 207 |
| **Everybody's Page** | 208 |
| **Recreation in Relation to the Health of the People.** VI.—In the Country | 209 |
| **Notes and News** | 210 |
| **General Charities** | 211 |
| **Scraps and Gleanings** | 211 |
| **The Student of Medicine.**—On German Students (*continued*)—Notes from the Schools—Appointments—Vacancies—Scholarships and Prizes—Pass Lists | |
| **Proposed Alterations at the Oxford Infirmary** | 211 |
| **Annotations.**—A Hopeful Departure—Sir Charles Russell and the Mad Doctors—The Oxford Infirmary: Heathen or Christian? | 212 |
| **The Practitioner's Mirror and Retrospect**— | |
| MEDICINE.—On Some Points in the Ordinary Process of Inflammation | 213 |
| SURGERY.—Diseases of the Fingers: A.—The Nails | 214 |
| THERAPEUTICS.—Hydrochlorate of Phenocoll | 215 |
| NEW DRUGS, APPLIANCES, AND THINGS MEDICAL.—The "Titan" Patent Soap | 215 |
| **Metropolitan Hospital Sunday Fund, 1891** | 216 |

EXTRA SUPPLEMENT—THE NURSING MIRROR.

| | |
|---|---|
| **En Passant.**—Trained Nurses' Club—Jewish Nurses—The Nurses' Co-operation—Short Items—Pension Fund Nurses—The Training of Nurses in Medical Electricity and Massage—Another Picnic | ci |
| **The Second Thousand** | cii |
| **Cape Colony** | civ |
| **Everybody's Opinion.**—St. John the Evangelist | civ |
| **The Princess of Wales Fund for Mrs. Grimwood** | civ |
| **Presentations** | civ |
| **For Reading to the Sick**—Memory | cv |
| **Appointments** | cv |
| **Wants and Workers** | cv |
| **Notes and Queries** | cv |
| **Off Duty.**—An Urgent Case (*concluded*) | cvi |
| **Death in Our Ranks** | cvi |
| **Amusements and Relaxation** | cvi |

